# Exploring RNA conformational space under sparse distance restraints

**DOI:** 10.1038/srep44074

**Published:** 2017-03-10

**Authors:** William R. Taylor, Russell S. Hamilton

**Affiliations:** 1 Computational Cell and Molecular Biology, Francis Crick Institute, London, NW1 1AT, UK; 2 Centre for Trophoblast Research (CTR), Department of Physiology, Development and Neuroscience, University of Cambridge, Cambridge, CB2 3DY, UK

## Abstract

We show that the application of a small number of restraints predicted by coevolution analysis can provide a powerful restriction on the conformational freedom of an RNA molecule. The greatest degree of restriction occurs when a contact is predicted between the distal ends of a pair of adjacent stemloops but even with this location additional flexibilities in the molecule can mask the contribution. Multiple cross-links, especially those including a pseudoknot provided the strongest restraint on conformational freedom with the effect being most apparent in topologically simple folds and less so if the fold is more topologically entwined. Little was expected for large structures (over 300 bases) and although a few strong localised restrictions were observed, they contributed little to the restraint of the overall fold. Although contacts predicted using a correlated mutation analysis can provide some powerful restrictions on the conformational freedom of RNA molecules, they are too erratic in their occurrence and distribution to provide a general approach to the problem of RNA 3D structure prediction from sequence.

The prediction of macromolecular structure from sequence data has been attempted by a large number of methods over many years for both protein and RNA structures. Both share the common problem that while local (secondary) structures can be predicted with reasonable accuracy, the assembly of these into a tertiary fold has proved to be particularly difficult—but not always impossible^1^,^2^,^3^,^4^. (See ref. 5 for a comparative protein/RNA review of basic principles and refs 6, 7, 8 for reviews of more RNA specific methods).

Recently, with the large numbers of known sequences and improved methods of covariation analysis, specific non-local correlations can be found that equate with positions in close proximity in the 3D structure. (See ref. 9 for a review). Despite this advance, the number of tertiary contacts that can be gathered from the majority of applications remain sparse and unreliable. This places greater importance on the modelling methods that are used to calculate the predicted structure and with sparse restraints many employ some form of coarse-graining so that the limited calculated restraints can be applied to the greatest effect^7^,^10^,^11^,^12^,^13^.

In an earlier study on globular protein structure prediction, it was found that, not unexpectedly, at least two restraints per secondary structure are needed to specify an almost unique fold^14^ and with this level of restraint, the fold of small globular proteins could be predicted from sequence^15^,^16^,^17^. The situation is more favourable for transmembrane structures (of the all alpha class) as these tend to have longer and fewer secondary structure elements that are more restricted in their range of packing compared to globular proteins^18^,^19^.

The problem of predicting RNA tertiary structure is helped by having large local secondary structure elements (stem-loops) that can be predicted well from sequence^20^,^21^, however, unlike protein structures in which a secondary structure can interact with several others, the interactions between stem-loops in RNA does not generally involve base pairing and hence are almost invisible to correlation analysis. Exceptions are found only in the non-local base pairs formed in pseudo-knots and in non-canonical base pair interactions^2^,^22^. This sparsity of non-local contacts is partly offset by the advantage that the signal from correlated positions, being derived from specific base pairing, is generally more reliable than contacts predicted from the more promiscuous amino acid interactions.

Given that the secondary structure elements in RNA are as large and almost as rigid as the helices in transmembrane proteins, if the rough rule of a few restraints per secondary structure applies, then a small number of non-local predicted contacts may be sufficient to specify a unique 3D structure. This could be tested by attempting to predict the 3D structure directly but except where the structures are relatively small with good restraints, success has been limited^1^,^4^.

A general application of prediction methods to problems with few restraints would be likely to reveal more about the performance of each method rather than the nature of the restraints as success would depend critically on the volume of conformational space that each method could explore in a reasonable time. Although we investigated folding for some smaller molecules, our main approach has been to invert the problem and instead we investigated how far the known structure could deviate (or be ‘denatured’) under random perturbations applied at a coarse-grained level. This allows us to apply the same protocol to all molecules, irrespective of their size or complexity, without being hindered by the limitations of a folding-based approach.

## Results

### Survey of the data

Rfam families containing a known structure and enough sequences for a clear correlation signal were further reduced by removing those that had little tertiary structure or were too large. (See Methods section for details of the selection criteria and Supplementary material for a description of the molecules and their restraints). The selected molecules were then divided into three groups that had 1–2, 3–4 and 5–6 predicted non-local interactions (Table 1).

Each RNA molecule was then simulated in a coarse-grained representation using two distinct simulation methods that operate at different granularities. SimRNA represents each nucleotide with five pseudo-atoms whereas SimGen uses a single point per nucleotide but these are tightly restrained in their secondary structures with random displacements and rotations applied at the secondary structure (stem-loop) level. Varying degrees of random perturbation were tested with each method using different restraint sets (described in detail in the Supplementary Material) and a level was selected for each method that produced a clear distinction between the restraint sets.

For each molecule, the restraints data-sets included: the observed base pairs defined by the ModeRNA server^23^, the base pairs predicted by the RNAfold program^24^ and contacts predicted by the Gremlin method^25^. While the former two methods provide a fixed set of base pairs, the contacts predicted by the latter method, which may or may not be base paired, constitutes a set of a few hundred contacts, each of which has a reliability score.

**Figure 1:**
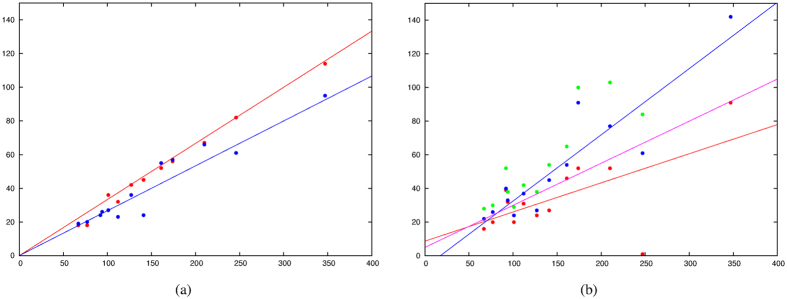
Expected base pairs per structure. (**a**) The number of observed basepairs using the ModeRNA server (Y-axis, blue dots) and the number of predicted base pairs using RNAfold (red dots) is plotted against the length of the molecule (X-axis). These lines provide a guide to the number of basepairs that should be expected using the correlated mutation analysis (Gremlin). (**b**) The number of contacts predicted by Gremlin is plotted (Y-axis) against the length of the molecule with a cutoff applied when the number of false predictions exceeded 1% (red dots), 2% (blue) and 5% (green). The red and blue lines are fitted to the 1 and 2% data and the magenta line is a compromise to exclude almost all the 5% error data points. This selects roughly the correct number of pairs without including too many false contacts which would disrupt the molecular simulations.

Although it might be possible to devise a weighting scheme that would allow all predicted contacts to be applied to varying degrees, to retain comparability with the fixed observed and predicted base pairs, we applied a cutoff on the number of top scoring contacts to use. As a first approximation and to provide direct comparability, we took the same number of contacts as base pairs predicted by RNAfold. When plotted against length, this closely follows the line L/3, where L is the length of the molecule (Fig. 1(a)).

To see if this cutoff could be improved, the frequency of incorrect contacts was plotted as a function of error rate (Fig. 1(b)). The L/3 cutoff approximates the two percent error rate but also encompasses the 5% error level for three molecules. Although 5% error may appear to be small, the majority of the correctly predicted contacts are ‘tied-up’ in secondary structure base pairs that contribute little to restraining the overall fold of the molecule and 5% error overall can equate with over 50% error in the remaining non-local contact predictions which are critical in determining the fold of the molecule. This level was therefore reduced to the linear approximation: *N* = 5 + *L*/4 (where *N* is the number of pairs to use for a molecule of length *L*), which is plotted in Fig. 1b where it runs between the 1 and 2% error rate.

**Figure 2:**
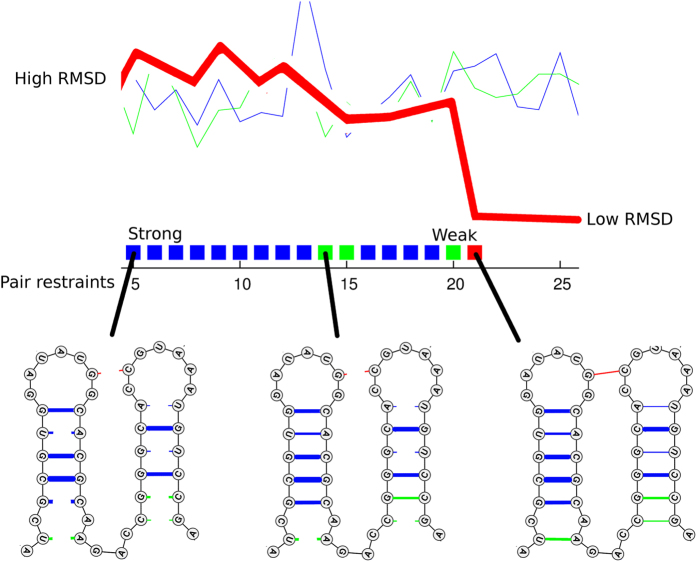
Example data plot. The data presentation used in Figs 3, 4, 5, 6, 7, 8 is illustrated with a simplified example based on Fig. 3c. The red line plots the Root Mean Square Deviation (RMSD) of a model from the known structure (Y-axis) against an increasing number of predicted pair restraints (X-axis). The restraints are introduced in ranked order of their predicted confidence, begining with the strongest pairs. Up to the expected number of basepairs, these are marked by a coloured square as: blue and green = local pairs (a green pair is not predicted by RNAfold) and red = non-local (i.e., between stem-loops). These are illustrated on the secondary structure diagram for a part of the molecule at three stages below the plot, with the strength of the restraint indicated by line thickness and coloured as above. In this example, the native structure is regained (drop to low RMSD) with the introduction of the non-local restraint (red) between the loops at the top of the diagram. (n.b., as this is a simplified example, using just part of the structure, the number of represented restraints do not exactly match those plotted). The actual constraints can be viewed for each molecule in the Supplementary Material using a similar representation. (See following Figure legends for details). The fine blue and green lines on the plot are control sets of restraints that do not include any non-local pairs and do not exhibit a drop in RMSD.

In the following sections, molecules are considered in turn in order of the number of predicted non-local constraints and for each molecule, a mean RMSD from the native structure is calculated over 10 models for each of the restraint sets. In order to see at what point the models become more restrained, this calculation is repeated for increasingly larger restraint sets. All restraints are treated equally in this progression, whether they connect local or non-local positions. This procedure is illustrated in Fig. 2.

### A single restraint

Four RNA molecules, including the three smallest, have only a single non-local restraint. All four can be represented as a Y-shaped topology with the termini locked in a double strand ‘trunk’ from which two stem-loops branch off. (See Supplementary Material for representations and a fuller description of the molecules and their restraints).

**Figure 3:**
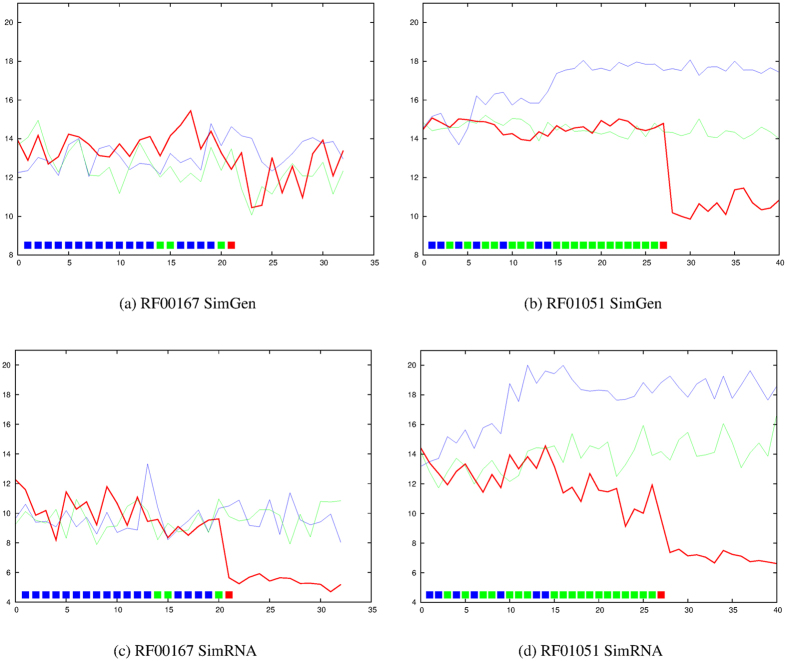
RF00167 (Purine riboswitch) and RF01051 (Cyclic di-GMP-I riboswitch). The average RMSD value from the native structure over ten models is plotted on the Y-axis for each molecule with increasing numbers of applied restraints (X-axis) using the predicted (Gremlin) data set (red line), the RNAfold base-pairs (green line) and the local known pairs as defined by the ModeRNA server (see Methods section for details). Plots are shown for two different simulation methods: SimGen (**a** and **b**) and SimRNA (**c** and **d**). The bar of coloured boxes near the bottom of each plot annotates each of the N/4 + 5 best predicted pairs (where N is the length of the molecule) as: blue = also found by rnafold, green = additional local pair found in addition to rnafold, red = true non-local pair and yellow = a false pair.

A link connecting the tops of the stem-loops can be expected to exert the greatest restriction on conformational freedom and two of the molecules, RF00167 (Figure S1(a),(b)) and RF01051 (Figure S1(c),(d)) have a restraint in this position. The degree of “denaturation” shows a sharp transition with both molecules at the point this restraint is applied for both simulation methods (Fig. 3).

**Figure 4:**
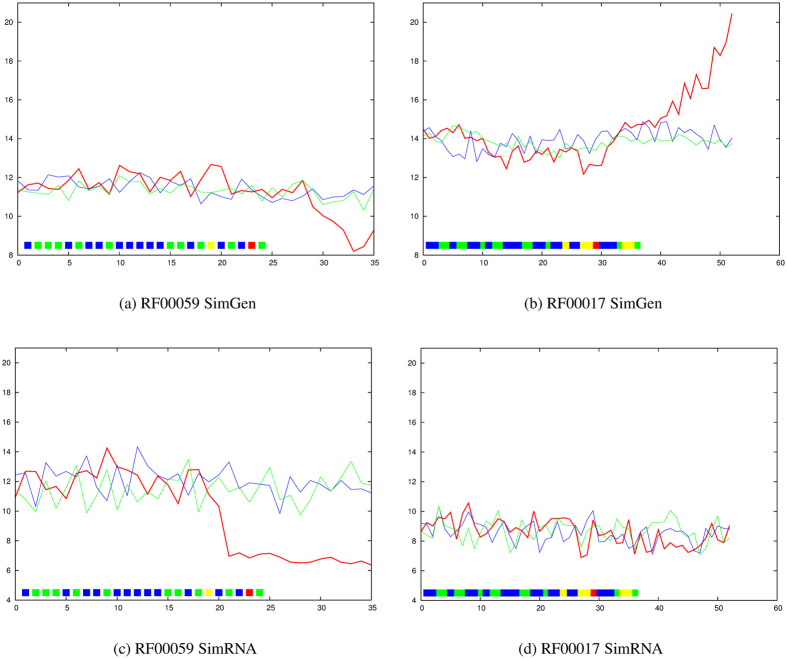
RF00059 (TPP riboswitch) and RF00017 (Metazoan SRP). The average RMSD value from the native structure over ten models is plotted on the Y-axis for each molecule with increasing numbers of applied restraints (X-axis) using the predicted data set. The lines and restraints (boxes) are coloured as in Fig. 2.

By contrast, RF00059 (Figure S2(a),(b)) and RF00017 (Figure S2(c),(d)) have a restraint at the fork in the “Y” and exhibit no transition associated with the application of the restraint (Fig. 4). Interestingly, the SimRNA profile has a transition associated with an incorrect prediction that links two positions at the top of the distal hairpins (yellow in Fig. 4(c) and Figure S2(a) and (b)).

### A few restraints

**Figure 5:**
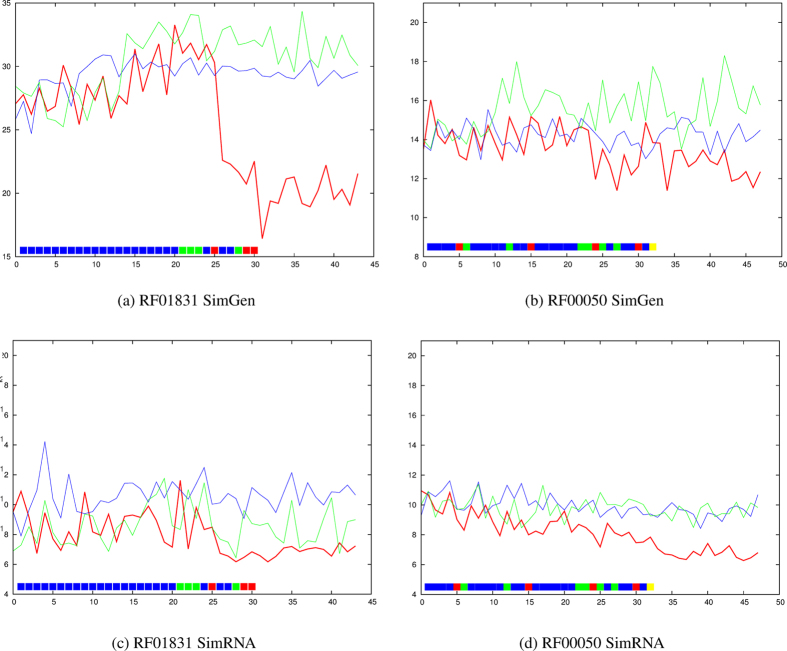
RF01831 (THF riboswitch) and RF00050 (FMN riboswitch). The average RMSD value from the native structure over ten models is plotted on the Y-axis for each molecule with increasing numbers of applied restraints (X-axis) using the predicted data set. The lines and restraints (boxes) are coloured as in Fig. 2.

In RF01831, the long-range tether provided by the three-basepair pseudo-knot (Figure S3(a),(b)) results in a clear transition to lower average deviation with the SimRNA method (Fig. 5(a)) and especially with the SimGen method (Fig. 5(c)).

The more complex scattering of long-range restraints in RF00050 (Figure S3(c),(d)) provide some restraint with both methods but with no marked transition (Fig. 5(b) and ((d) for SimGen and SimRNA, respectively).

**Figure 6:**
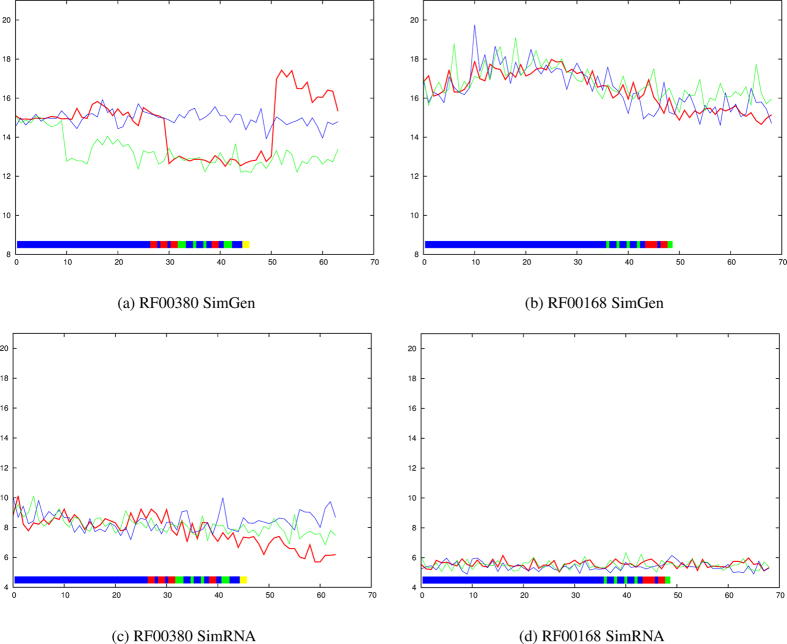
RF00380 (ykoK leader) and RF00168 (Lysine riboswitch). The average RMSD value from the native structure over ten models is plotted on the Y-axis for each molecule with increasing numbers of applied restraints (X-axis) using the predicted data set. The lines and restraints (boxes) are coloured as in Fig. 2.

The larger molecules, RF00380 (Figure S4(a),(b)) and RF00168 (Figure S4(c),(d)) were particularly resistant to “denaturation” by SimRNA and the slightly better retention of the native conformation with increasing numbers of restraints may simply reflect a general reduction in the mobility of the molecule (Fig. 6(b) and (d)).

SimGen, by contrast was able to induce larger movements, showing a clear transition associated with the imposition of a cluster of three long-range restraints in RF00380 (red in Fig. 6(a) and Figure S4(a),(b)). As a greater number of restraints are introduced, a reverse transition to higher RMSD is seen as erroneous contacts are included. In RF00168 a smaller transition is seen against an already high RMSD background contributed by unrestrained parts of the molecule (Fig. 6(b)).

### Several restraints

The RNA molecules with the greatest number of non-local restraints, not unexpectedly, include the two largest structures (RF00010 and RF00028) but also RF00162 with only 94 bases and the RF00234 molecule which is of intermediate length.

**Figure 7:**
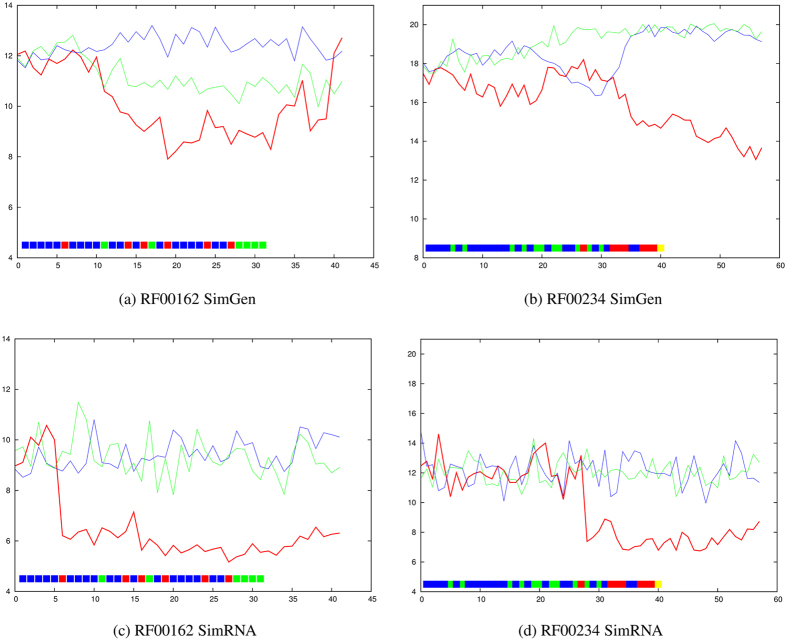
RF00162 (SAM riboswitch) and RF00234 (glmS glucosamine-6-phosphate activated ribozyme). The average RMSD value from the native structure over ten models is plotted on the Y-axis for each molecule with increasing numbers of applied restraints (X-axis) using the predicted data set. The lines and restraints (boxes) are coloured as in Fig. 2.

RF00162 has a cruciform topology (Figure S5(a),(b)) which, combined with its smaller size, gives it some resistance to large-scale denaturation motions. Despite this, a clear transition can be seen with both simulation methods. With SimRNA (Fig. 7(c)) the transition is sharp and occurs with the imposition of the first restraint in the pseudo-knot, whereas with SimGen, the transition is spread over the incorporation of all three basepairs in the pseudo-knot plus an additional non-local restraint (Fig. 7(a)).

A similar distinction can be seen with RF00234 which has a gradual transition with SimGen (Fig. 7(b)) compared with an immediate shift with SimRNA on the imposition of the first (highest-scoring) non-local restraint (Fig. 7(d)). This link between positions 23 and 11, tethers a major stem-loop close to the 5′ terminus (top in Figure S5(c),(d)) with additional restraints adding further tethers at both termini.

For the two largest molecules (RF00010 and RF00028) some retention of the native conformation can be seen with the application of the predicted restraint set (Figure S11(c) and Figure S11(d)) but given the complexity of analysing these differences and the increased computational demands, a full set of incremental restraint removal results was not calculated.

### Folding experiments

For the smaller molecules that exhibited some difference between the known restraint set and the predicted restraint set, a series of folding simulations was performed starting from an extended (circular) starting configuration. Six molecules (RF00059, RF00162, RF00167, RF00234, RF01051, RF01831) were folded with both simulation methods using both the known restraint set (which has only local pairings that can be expressed in bracket notation) and the predicted restraint set (which includes some non-local pairings) and the deviation from the native structure measured as a simple RMSD value (Table 2).

Comparing the RMSD values between the runs with known and predicted restraints, it can be seen that all the simulations, bar one, attain a lower RMSD value when the predicted restraints are applied. Overall however, the values obtained tend to be higher than those seen in the denaturation experiments and examination of selected models suggests that a contribution to this effect comes from a less accurate orientation of the secondary structures about their axis. Unlike the protein *α*-helix, there is no hydrophobic moment to guide their orientation and the typical location of the non-local predicted restraints in loop regions has only an indirect influence on relative stem-loop phasing.

Comparing the results from the different simulation methods, simRNA produced models with lower RMSD values but with little gain made by trading a longer run-time for fewer models. The simGen method showed little gain for RF00167, reflecting its behaviour in the denaturation experiments where the intimate packing of the two stem-loops was not reproduced. Despite a large transition in the denaturation experiments, no gain was observed for RF01831 with large natural conformations of the poorly restrained termini being the apparent cause.

### RNA Puzzles

An additional source of molecules to test was found in the collection employed in the RNA Puzzle experiment^3^. Of these 14 molecules, five had sufficient sequence homologues to be analysed by the Gremlin program but after its redundancy filters had been applied, only three remained. (Puzzles: 3,4 and 6. See Supplementary Material for details of the data). Puzzle-4, however, bar a 25 nt. insert, is 90% identical to the SAM riboswitch (RF00162) analysed above. Puzzle-3 (86 nt.) has a few predicted pairings that are not directly part of a base-paired ladder but do not constitute true non-local restraints. Puzzle-6 (168 nt.) has several true non-local restraints but has a structure of challenging size and complexity.

**Figure 8:**
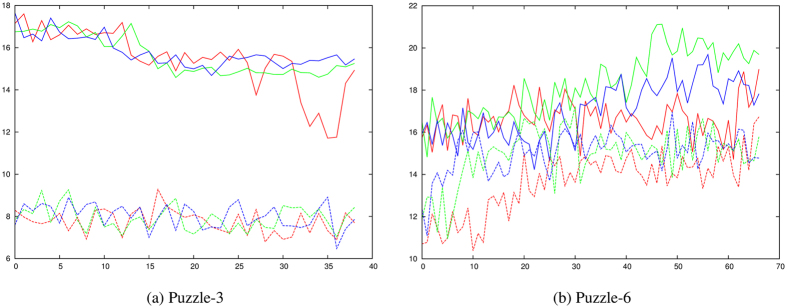
Denaturation plots for two RNA-Puzzle examples. (**a**) Puzzle-3 (glycine riboswitch, PDB code: 3OWZ) and (**b**) Puzzle-6 (adenosylcobalamin riboswitch, PDB code: 4GXY). The RMSD values for each simulation are plotted as for the Rfam examples described above except that the SimGen results are plotted with full lines and the SimRNA results are plotted with dashed lines. No step transitions are observed for either molecule.

When run through the denaturation protocol with increasing numbers of predicted restraints (as described above for the Rfam families), neither of the remaining Puzzle examples exhibited a consistent transition induced by the predicted restraints (Fig. 8).

## Discussion

In this work, we have taken a purely structural view in assessing the effect of the restraints available through an analysis of correlated mutations on the conformational freedom of a wide variety of RNA molecules.

### Summary of the results

#### Denaturing simulations

For all the molecules considered, we were able to run a series of ‘denaturing’ simulations that tested, for restraint sets of different sizes, how far the structure could be displaced from the native starting structure.

The application of a single (or single cluster) of restraints can provide a powerful restriction on the conformational freedom of an RNA molecule but only when it forms a cross-link between the distal ends of a pair of adjacent stem-loops (RF01051, RF01831) and even with this location additional flexibilities in the molecule can mask the contribution (RF00167). Restraints at the proximal end of stemloops contribute little to restrict their freedom (RF00017, RF00059).

Where there are multiple stem-loops, of which only a pair share a favourable cross-link, then the overall RMSD is a poor measure of any restraint as the “noise” generated by the free loop(s) can partly mask conformational restriction in other parts. Plotting the RMSD along the sequence can be used to identify restrained segments (RF00380, RF00168).

Multiple cross-links, especially those including a pseudoknot provided the strongest restraint on conformational freedom (RF00050, RF00162, RF00234). The effect is most apparent on a topologically simple fold (RF00234) and less so if the fold is more topologically entwined (RF00162). The restriction of the restraints can be slightly increased by imposing an idealised double-strand geometry on the pseudoknot but this would require a prediction of the pseudoknot.

Little was expected for large structures (RF00028, RF00010) and although a few strong localised restrictions were observed, they contributed little to the restraint of the overall fold.

#### Folding simulations

For several of the smaller molecules we were also able to test the reverse process of how close the models could approach the native conformation starting from a circular extended conformation.

In all of these simulations, bar one, the best models were obtained using the set of constraints predicted by correlated mutation analysis. However, overall the RMSD values obtained were larger than those in the denaturation experiments. While this gap may simply reflect that all the simulations require a longer running time, it was apparent that a sizeable contribution to this difference came from the relative axial orientation of the their stem loops. This is a degree of freedom that is not strongly constrained by the application of restraints that are largely located in the loop region of the stemloops.

## Conclusions

In our analysis of the conformational freedom of a variety of RNA molecules under sparse non-local restraints derived from correlated mutations, we have shown that the degrees of restraint range from almost nothing up to a level that is sufficient to specify a unique fold in the simpler molecules. However, unlike the equivalent situation for protein structure, which often form a dense interconnected network of predicted contacts, the sparse network seen for RNA molecules cannot generally be expected to provide a unique solution for the overall 3D fold.

With protein structure, the amino acids observed at any given position are able to exert evolutionary selection pressure on their neighbours in the three dimensional structure with which they make direct, or even indirect, contacts. By contrast, the specific base pairing of the nucleotides in RNA “locks” most of their capacity for interaction into bound pairs leaving little with which to influence the selection pressure on other nearby positions, even adjacent base pairs. If there were a significant non-base paired component, then the predicted interaction network within and between stemloops would include more than one link per base and the 1:1 basepaired nature of the predicted contacts seen in ladders would not be observed. This lack of ‘cross-talk’ is a bonus for predicting secondary structure but hinders tertiary prediction.

When the base pairing interactions contribute to a non-local pseudoknot structure, then there is a strong restriction on the conformational freedom of the molecule but this is more of an exceptional situation, occurring in perhaps one location in less than half the molecules examined. In addition, the prediction of a pseudoknot can also be made from a more conventional analysis of base pairing specificity^22^,^26^ and is not information that can be gained uniquely from an analysis of correlated mutations.

Non-local interactions mediated through non-canonical base pairing interactions are much more difficult to predict using conventional sequence analysis but sometimes can be strongly predicted using a correlated mutation analysis. These have a greater capacity to provide a more general source of restraint, however, they still do not approach the capacity of amino acid promiscuity in their ability to provide restraints. From the ranking of those observed in this work, they are also predicted with less certainty than base pairings in double-stranded secondary structure.

In summary, although contacts predicted using a correlated mutation analysis can provide some powerful restrictions on the conformational freedom of RNA molecules, they are too erratic in their occurrence and distribution to provide a general approach to the problem of RNA 3D structure prediction from sequence on their own. Their power may be best exploited in combination with more general experimental methods such as chemical, enzymatic or NMR^27^,^28^,^29^,^30^,^31^ along with conventional base pairing analysis for regions that have no correlation signal. All these sources of restraints can then be weighted and synthesised in any combination of the coarse-grained or *ab initio* modelling methods reviewed in the Introduction.

## Methods

### Data Selection

RNA sequence alignments were taken from the Rfam databank (http://rfam.xfam.org/)^32^. As the latest release of the databank no longer holds the full multiple sequence alignments, these were regenerated with the Infernal program^33^, using the hidden Markov model provided in the databank. The number of sequences in each family is given in Table 3 (col. 3). As some of these sequence collections are very large, they were reduced by the removal of the most similar sequences to around or below 5000 representatives using the program Mulsel^34^ (Table 3, col. 4).

The reduced alignment was then passed to the Gremlin program^35^ where it was further reduced using the program’s internal default cutoff, resulting in the number of sequences shown in Table 2 column 5. The program calculates the number of sequences/length (Table 3 col. 6) and although a ratio of 5 is recommended, for RNA, the quality of the data remained reasonably clear of “noise” even with a ratio below 2.

**Figure 9:**
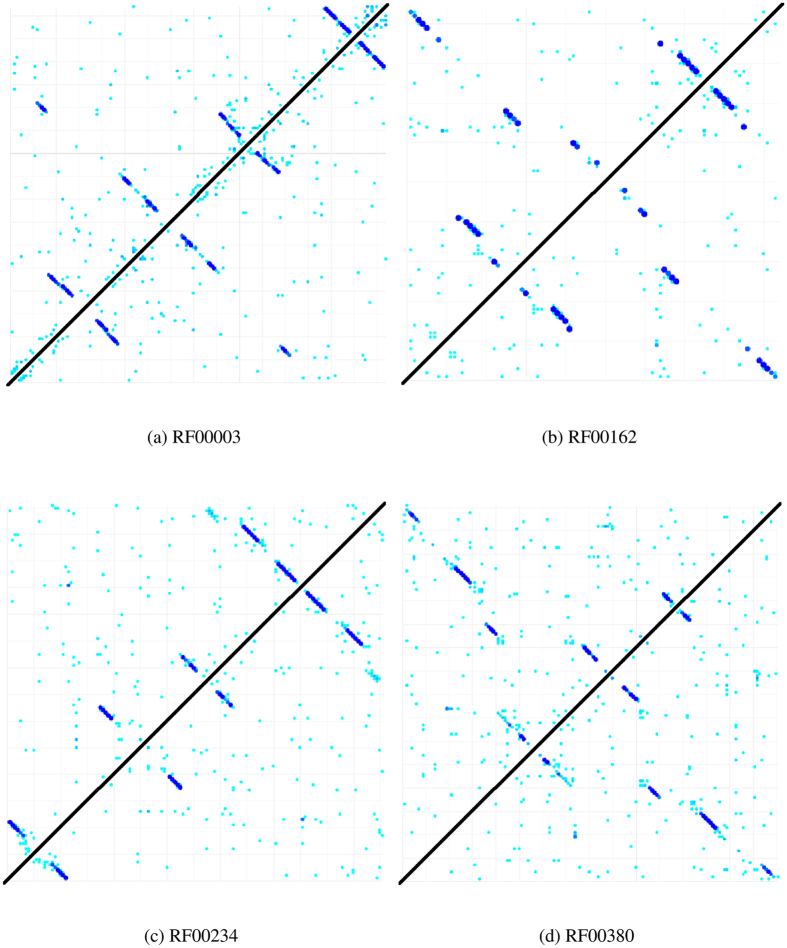
Predicted contact maps. The contacts predicted by the Gremlin method are shown for four molecules, two of which have a high sequence/length ratio: RF00003 = 14.1, RF0162 = 10.7 (**a**,**b**) and two of which have a low ratio: RF00234 = 1.9, RF00380 = 1.1 (**c**,**d**). The order of magnitude difference in their levels of coverage is not strongly reflected in the quality of the plots. (Dark blue = strong contact, light blue = weak).

In Fig. 9, four predicted contact sets are compared for molecules with very different ratios. It is clear from the plots that there is very little qualitative difference between those with a high sequence/length ratio and those with a low ratio. In addition, the transition from strong to weakly predicted contacts remains reasonably sharp.

### Restraint sets

Three sets of basepair restraints were used. Two of these were based on explicit base pairing and contain only local pairs which are defined as those that can be expressed in a nested bracket notation. The third, based on the analysis of correlated mutations, can include any pairing.

#### Observed basepairing

Observed base pairs were defined on the full known structure by the ModeRNA server (http://iimcb.genesilico.pl/modernaserver/submit/analyse/)^23^.

Any segments that were missing from the PDB structure were also added using the RNA modelling program available at this site^36^.

#### Predicted basepairing

Base pairings were predicted for the Rfam probe sequence (corresponding to that found in the PDB structure) using the RNAfold program from the Vienna Package (www.tbi.univie.ac.at/RNA/)^21^,^24^.

#### Predicted contacts

Correlated mutations were predicted using the Gremlin server (http://gremlin.bakerlab.org)^25^,^35^. The reduced Rfam alignment was entered which was then further reduced internally by the server giving the reliability factor (sequences/length) shown in Table 3, column 6.

### Coarse-grained simulation

#### SimGen

SimGen^37^,^38^ is a simple coarse-grained simulation method that uses only a phosphate backbone representation but ladders of three or more consecutive base pairs were defined as secondary structure elements (SSEs) and represented as tubular segments. The internal geometry of these secondary structures were continually refined towards ideal geometry and the SSEs were displaced and rotated randomly by a small factor on each cycle of the simulation.

During the simulation, phosphate/phosphate virtual bond lengths were continually refined towards an ideal length of 6 Å and any non-bonded phosphate/phosphate contact under 10Å was repelled. To restrict any unnatural conformations being adopted in the loop regions, local distances between 10 consecutive phosphates were also refined towards their native values. This was implemented by applying a small shift on each cycle to restore any distance in this window towards its starting value. This was only applied if the current distance was less than 18 Å allowing large changes in conformation to be accepted. Although this applies some restraint on the global fold, shifts of up to 50Å away from the native structure were still observed in the distal portions of stem-loops.

Restraints from the predicted or observed base-pairings were all applied in the same way whether the pair was a relatively local assignment in a stem-loop or an isolated non-local interaction. Since the target distance is between phosphates, it makes no difference if the base pairing is canonical or non-canonical. These distances were refined using the built-in regularisation routine in SimGen that shifts the pair of atoms towards their target separation of 17Å by a fixed step on each cycle of the simulation. The refinement was only applied to pairs with a separation greater than the target distance and in the final model had the effect that roughly 20% exceeded the target by 1 Å, 2% by 2 Å and it would be very rare for a restrained pair to exceed the target by more than a few Ångstroms. (See ref. 38 and Supplementary Material for details).

**Figure 10:**
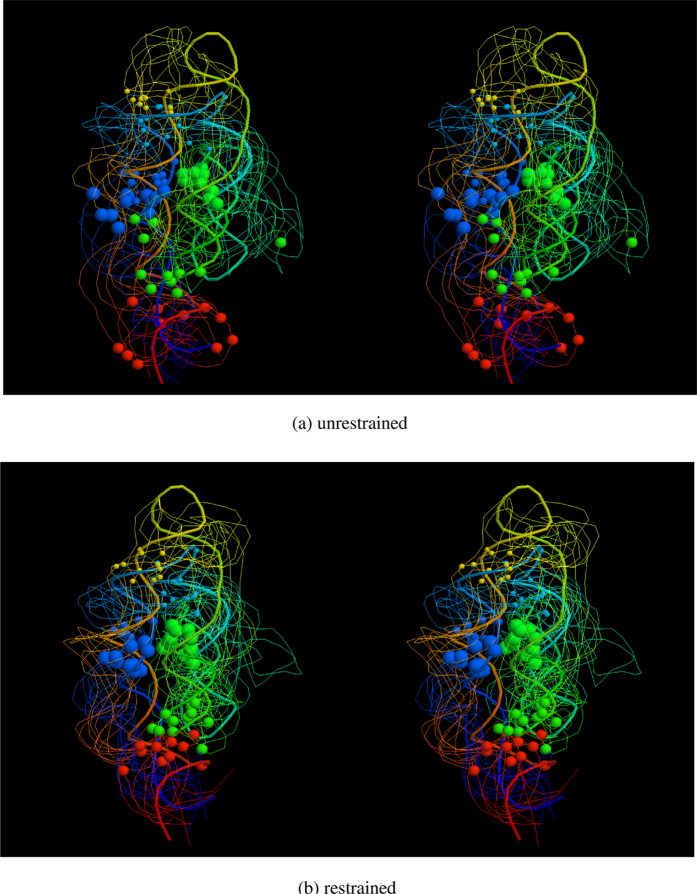
Typical simulation results. The phosphate backbones of the final ten SimGen models for RF00380 (fine lines) is shown as a stereo pair, coloured from blue (5′) to red (3′). The PDB structure (2qbz) on which they have been superposed is similarly drawn with a thicker line. Four pairs of non-local restraints are depicted as balls of different sizes on the phosphate atoms that they connect with a diameter reflecting their ranked order (largest = strongest). (**a**) shows the resulting models when the restraints are not imposed and (**b**) shows the resulting models when they are. The difference in RMSD can be seen in Fig. 6(A) as a drop fro 15.0 to 12.5 Å, where the results are discussed. The orientation is the same as Fig. S4(A).

To establish the degree of perturbation to use for the tests described in the Results section, a simulation of 1000 cycles was carried out 10 times using each of the three restraint sets described above. Five separate simulations were run with the random displacement applied to SSE elements varied from zero up to 0.1 Å(translation) and up to 0.1 rad. (rotation). A set of models is shown in Fig. 10 for a middle-sized molecule with a few restraints after a run with the maximum level of perturbation.

It can be seen from the RMSD plots of these data in the Supplementary Material (“Testing the perturbation level” section, Figures S8 and S9) that a good separation between the Gremlin and other data sets is generally attained before the maximum and a value of 0.08 Å (position 4 on the plots) was used.

#### SimRNA

SimRNA^13^,^36^ is a coarse-grained simulation method in which each nucleotide is represented by five pseudo-atoms: two in the backbone (including the true phosphate and C4′ sugar atoms) and three representing the base as a triangle. Restraints can be applied between any pairs of atoms but by specifying a secondary structure definition (in bracket notation), base pairing restraints are created between the two pairs of atoms linking the triangular pseudo-bases.

In addition to these, predicted contacts were added but these used the additional pseudo-base centroid atom (MB) as it cannot be established from a predicted contact whether the pairing is canonical, non-canonical or even just base-stacking. These restraints were maintained by a deep square-well extending from 5–10 Å separation using the command-line: WELL A/16/MB A/39/MB 5.0 10.0 5.0. To allow the restraint to become re-established should it escape from the well, a flanking V-shaped slope was also added around the well as: SLOPE A/16/MB A/39/MB 5.0 10.0 1.0. (Both example commands specify a link between positions 16 and 39 in chain ‘A’).

The degree of random perturbation can be controlled both through a simulation ‘temperature’ and the length of the run. Trials were made with various settings of these parameters to find a combination that would generate substantial displacements but not disrupt the integrity of the secondary structure components. We found that a temperature gradient starting from 2.0 and cooling to 1.0 at the end of the run met these requirements for simulation lengths ranging from 1000 to 10,000 cycles.

SimRNA was also run ten times for each of five run-lengths on each molecule using exactly the same restraint datasets as described above for SimGen.

### Analysis of changes

Changes in the models were monitored by the root-mean-squared-deviation (RMSD) from both the native structure and the mean model structure.

#### Euclidean RMSD

The conventional RMSD value based on the rigid-body optimal superposition between two structures was measured between equivalent phosphate atoms. This was reported as a mean value over the 10 models calculated with each parameter and restraint set combination.

#### Distance RMSD

The shift of a subdomain away from its position in the native structure can lead to a large change in RMSD yet the internal structure of the domain may be well preserved. To focus more on the aspects that change under such a displacement, we used the distance based RMSD (dRMSD) which is based on the sum of the squared deviations in each pairwise distance between phosphate atoms.

#### Maximum dRMSD

The mean dRMSD over the 10 models may not reflect the full extent to which the structure is free to deviate and to capture this, we also used the maximum observed value to calculate an overall dRMSDmax.

**Figure 11:**
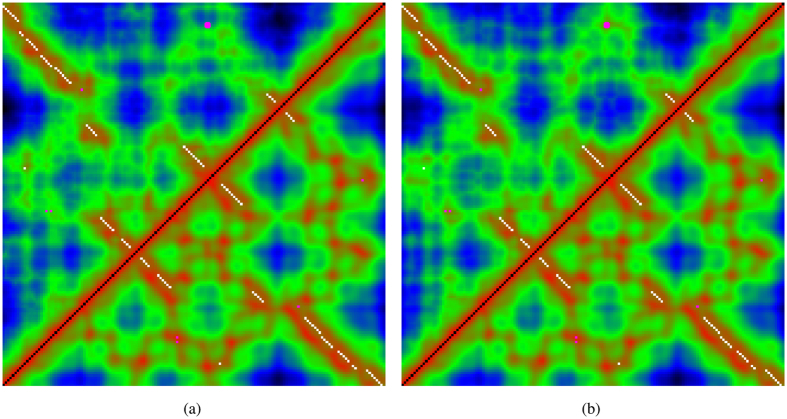
Maximum-distance matrices for RF00380. The pairwise distances between phosphates in RF00380 are plotted as a distance matrix coloured red from the closest pairs through green and blue to black for the most widely separated pairs. The white dots mark local base paired positions and a few magenta dots mark non-local pairs. The lower-right half of the matrix shows the native distances and the top-left show distances from (**a**) the maximum distance seen in the ten models generated using the Gremlin predicted contact restraint set and (**b**), the observed basepairs. Some loss of retention of contact can be seen near the middle of the top edge associated with the imposition of a restraint between positions 87 and 152 (marked as an enlarged magenta dot). This pair of bases can be identified in the lower part of Fig. 10 as the medium sized red and green balls.

A representation of the models shown in Fig. 10 as the distance matrix from which the dRMSDmax is calculated, is shown in Fig. 11.

### Folding simulations

The two simulation methods (SimGen and SimRNA) were also employed in a limited number of folding simulations, both starting from the default extended circular conformation generated by SimRNA. The SimRNA simulations used the same parameters as the unfolding experiments described above but with a longer run times of 100,000 to 1,000,000 steps. The SimGen runs retained the same run length. As the same computer time was allocated to each method, SimGen constructed roughly ten times more models than the SimRNA runs.

All the simulations were run with both the known (local) restraint set and the Gremlin restraint set using *N/*4 + 5 pairs (as determined in the Results section). As such, these simulations are not true *ab initio* folding experiments as they use the known secondary structure definitions and were evaluated only by their RMSD to the native structure. Their purpose was to establish if native-like conformations can be accessed more frequently when the predicted restraints are employed.

## Additional Information

**Accession Codes**: All source codes and data used in this work can be accessed at: https://github.com/darogan/ExploringRNAConformationalSpace.

**How to cite this article:** Taylor, W. R. and Hamilton, R. S. Exploring RNA conformational space under sparse distance restraints. *Sci. Rep.* **7**, 44074; doi: 10.1038/srep44074 (2017).

**Publisher's note:** Springer Nature remains neutral with regard to jurisdictional claims in published maps and institutional affiliations.

## Supplementary Material

Supplementary Information

## Figures and Tables

**Table 1:**
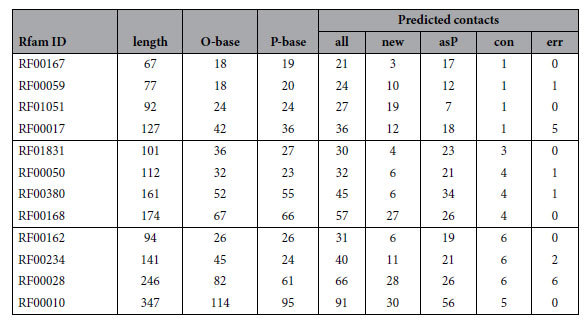
Predicted and observed basepair counts are tabulated for each of the RNA molecules used in the study (Rfam ID, col. 1 and length, col. 2).

Column 3 (O-base) is the number of observed basepairs as defined by the ModeRNA server, column 4 (P-base) is the number of basepairs predicted by the RNAfold program and column 5 (all) is the number of contacts predicted by Gremlin (falling above the cutoff defined in the text). The latter number is broken down into novel contacts (new, col. 6), those matching RNAfold (asP, col. 7), followed by the number of non-local contacts (under 21 Å) and false contacts (over 21 Å) as con and err in columns 8 and 9.

**Table 2:** RMSD values after folding with restraints.

Method	RF00059	RF00162	RF00167	RF00234	RF01051	RF01831
N	33534	32590	34012	32560	32562	32654
Rp	12.06	12.30	13.90	18.71	12.86	16.43
simGen						
N	32565	32554	32686	32540	32557	32580
Ro	13.03	16.29	14.15	20.52	16.03	16.10
N	4946	4000	5857	2471	3496	3646
Rp	8.46	11.36	5.52	16.46	9.24	10.50
simRNA’						
N	5593	4452	6484	2955	4860	4080
Ro	10.46	14.60	8.10	22.80	16.37	14.88
N	472	365	548	228	334	326
Rp	8.27	11.47	6.31	13.48	8.84	12.17
simRNA”						
N	542	407	605	275	447	375
Ro	12.88	15.91	10.26	23.32	18.43	15.70

For each of the molecules that exhibited an RMSD difference in the denaturation experiments between the known (local) restraints and the predicted restraints, a folding simulation was tested starting from an extended circular conformation using both the SimGen and SimRNA methods, with the latter run for lengths of 100,000 and 1,000,000 steps (simRNA’ and simRNA”, respectively).The rows flanking each program name indicate the number of models (N) constructed and the mean RMSD of the ten models closest to the native structure. Those above (Rp) used the predicted set of constraints (which includes some non-local pairs) while those below (Ro) are from the known structure, including only local pairs (defined as those represented in nested bracket notation). In all simulations (bar one), the Rp values is less than the Ro value.

**Table 3:** RNA families with a known structure are identified by their Rfam database identity number (Rfam ID, col. 1) and their PDB identifier (pdb ID, col. 2).

Rfam ID	pdb ID	Number of sequences			Sequences	
		Start	Filter	Used	Per length	Notes
RF00003	3pgw	15770	5072	2310	14.09	2 non-local but not compact
RF00004	2lkr					not enough seqs overlap pdb
RF00005	3wfs					little tertiary str (tRNA)
RF00008	2qus					not enough seqs
RF00010	3q1r	3147	3002	1516	4.39	many non-local contacts
RF00011	2a64					not enough seqs
RF00017	2j37	12763	9178	761	6.40	1 non-local contact
RF00026	4n0t					no tertiary fold
RF00028	1grz	3290	3290	647	2.86	many non-local contacts
RF00029	1kxk					no tertiary fold
RF00050	3f30	4382	4382	683	6.10	a few non-local contacts
RF00059	3d2x	1495	1480	565	7.34	1 non-local contact
RF00114	2vaz					partial pdb
RF00162	2gis	4906	4891	953	10.71	several non-local contacts
RF00167	4fe5	2590	2590	474	7.08	1 non-local contact
RF00168	3dj2	2900	2866	686	3.97	3 non-local contacts
RF00174	4gma	4893	4600	1325	7.09	truncated alignment
RF00234	3l3c	983	959	271	1.94	a few non-local contacts
RF00374	1s9s					no tertiary fold
RF00380	2qbz	1500	1495	192	1.19	a few non-local contacts
RF00504	3oxm	4603	4603	871	10.13	no non-local contacts
RF01051	3ucz	2232	2196	1075	13.11	1 non-local contact
RF01073	2lc8	7454	2898	660	11.19	single stemloop (with ext.n)
RF01734	3vrs	1426	1426	428	8.23	too small
RF01831	3suy	611	608	178	1.80	a few non-local contacts
RF01852	3hl2	2184	2184	468	5.37	no non-local contacts
RF01998	4fb0					partial coverage
RF02001	4fb0					partial coverage
RF02095	2l3j					no tertiary fold

The number of sequences collected in Rfam (start, col. 3) is reduced by removal of the most similar sequences (filtered, col. 4) and again in Gremlin (used, col. 5) which calculates the number of sequences per length (col. 6) as a guide to the quality of the predicted contacts. Any value below 1 was not used. Some brief comments follow on the sequences, structure and predicted contacts (notes). For the latter the term “non-local” means that the base pair is not included in the nested-bracket representation of the observed basepairs calculated by the ModeRNA server, given the full PDB structure.
