# Supplementary material for: Exploring RNA conformational space under sparse distance restraints
Source: Sci Rep. 2017 Mar 10;7:44074. doi: 10.1038/srep44074 (PMC5345030; doi:10.1038/srep44074)
Supplement: Supplementary Information [file srep44074-s1.pdf]

SUPPLEMENTARY MATERIAL for Exploring RNA conformational space under sparse distance restraints

William R. Taylor[†*] and Russell S. Hamilton[‡]

[†] Computational Cell and Molecular Biology,
Francis Crick Institute,
1 Midland Road, London NW1 1AT, UK

[‡] Centre for Trophoblast Research (CTR),
Department of Physiology, Development and Neuroscience,
University of Cambridge, Cambridge, UK

[*] corresponding author: william.taylor@crick.ac.uk

Keywords:
non-canonical base pair interactions;
base pair covariation;
Coarse-grained simulation.

January 19, 2017

Description of the molecules and their restraints

The length of the molecule in nucleotides (nt) and the number of non-local restraints (const) is given in parentheses as "(nt,const)" on each subsection heading as the molecules are considered in turn below. For each molecule, these restraints are visualised as a phosphate backbone representation along with a smoothed trace. The latter was included just to facilitate visualisation and the smoothed backbone played no part in the calculations or the comparison of the molecules.

Purine riboswitch: RF00167 (67nt, 1const)

The smallest molecule, RF00167, has a single non-local restraint at position 20 in the ranked list of contacts. This falls outside the cutoff defined by the number of RNAfold predicted base pairs but is just inside the linear cutoff defined above. As can be seen in Figure S1(a), this restraint is well placed to restrain the motion of the two stem-loops where it forms a cross-link between their loops.

Cyclic di-GMP-I riboswitch: RF01051 (92nt, 1const)

RF01051 also has a single cross-link connecting the loops at the top of the forked stemloops. In contrast to RF00167 in which the base pairs in the stemloops were well predicted, the RNAfold predictions in RF01051 suffer a major slip in the longer stemloop but are correctly predicted by the correlation analysis which include no false contacts.

TPP riboswitch (THI element): RF00059 (77nt, 1const)

Like RF00167, RF00059 has a single restraint that was not included with the predicted base pair cutoff but was with the linear cutoff. This restraint, however, lies close to the base of the fork in the Y-shape and, as can be seen more clearly in the smoothed representation (Figure S2(b)), could be considered as an outlying local link in the stemloop on the right.

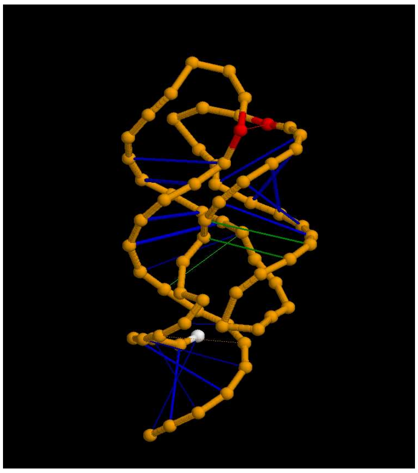

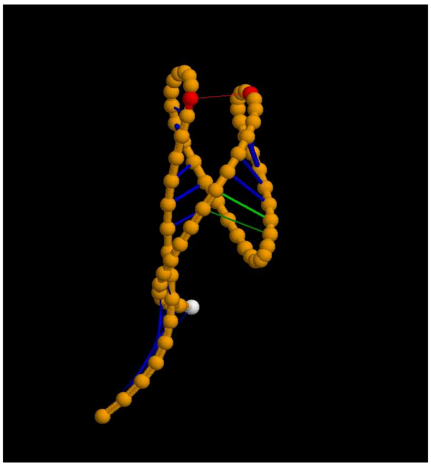

(a) RF00167 backbone

(b) RF00167 smoothed

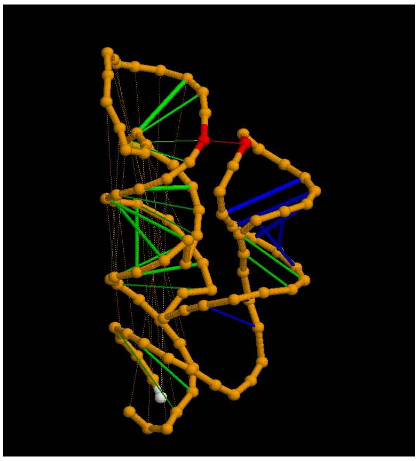

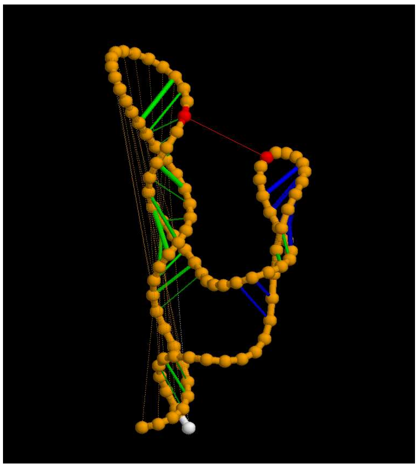

(c) RF01051 backbone

(d) RF01051 smoothed

Figure S1: **RF00167 (Purine riboswitch) and RF01051 (Cyclic di-GMP-I riboswitch)** phosphate backbones are shown in panels (*a*) and (*c*) with smoothed backbones in panels (*b*) and (*d*), respectively. Restraints are coloured: blue where a Gremlin predicted contact corresponds with an RNAfold predicted base pair, green for a correctly predicted contact (under 21 Å), yellow for an incorrect contact and red for a topologically non-local contact (not part of any extended ladder). For the latter pair of links, the phosphate atoms are also given the same colour. The thickness of the link indicates the position of the pair in the ranked list of contacts (thickest = highest). The base pairs predicted by RNAfold are marked by feint dashed orange lines. The white phosphate marks the 5′ terminus.

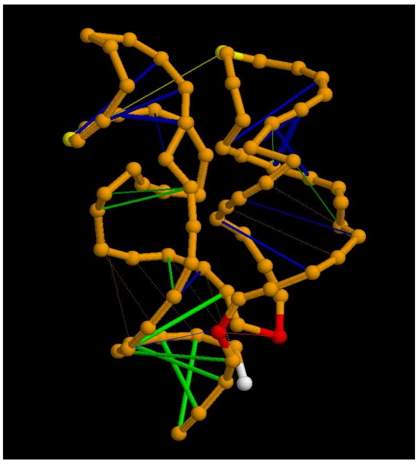

(a) RF00059 backbone

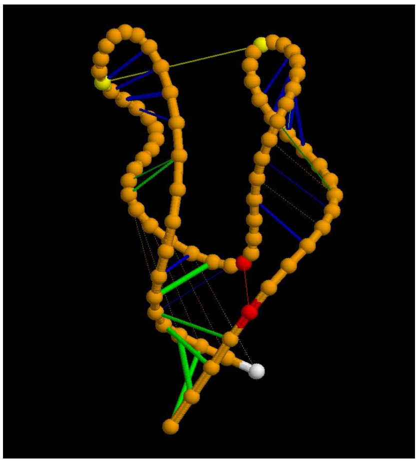

(b) RF00059 smoothed

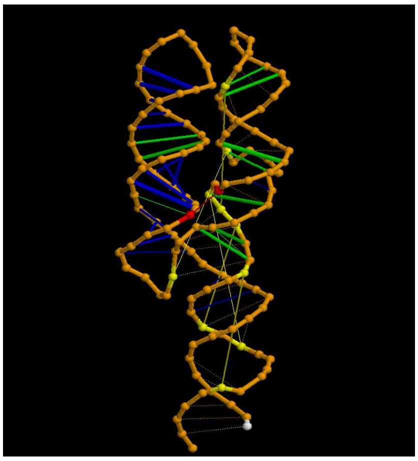

(c) RF00017 backbone

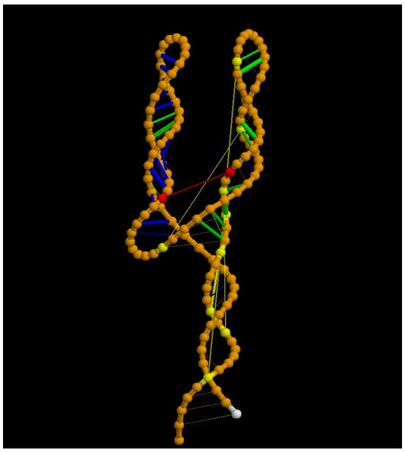

(d) RF00017 smoothed

Figure S2: **RF00059 (TPP riboswitch) and RF00017 (Metazoan SRP)** phosphate backbones are shown in panels (*a*) and (*c*) with smoothed backbones in panels (*b*) and (*d*), respectively. Restraints are coloured as in Figure 2.

Metazoan signal recognition particle: RF00017 (127nt, 1const)

The cross-link in RF00017, like RF00059, also lies at the fork of the Y-shape, however, it is a true non-local connection and cannot be considered part of any extended base pair ladder. At ranked position 33, it is included in both the sets of restraints that were tested but so also were a number of false contacts (yellow in Figure S2(c) and (d)). The double stranded segment formed by the termini, despite having several base pairs correctly predicted by RNAfold, contains no predicted Gremlin contacts. This was a result of poor sequence coverage at the $5'$ end of the multiple sequence alignment.

THF riboswitch: RF01831 (101nt, 3const)

The RF01831 molecule is topologically a single hairpin that has been doubled-over in the middle. In the known structure, the termini are single stranded and separated, however, this is the result of an exchange of termini between adjacent molecules in the crystal and the termini are predicted to be base paired by both RNAfold and Gremlin. As such, the wide spacing between them was not counted as a false prediction. Where the mid-loop approaches the termini, a run of four base pairs is formed between segments 41–44 and 94–91, forming a classic pseudo-knot. In this region, a cluster of three contacts is predicted by Gremlin. (Figure S3(a)).

ykoK leader: RF00380 (161nt, 4const)

The previous two topologies are combined in RF00380 which forms a doubled-over Y-shape with predicted non-local contacts between the trunk and one of the branches. Only one false contact is predicted but at 26 Å it lies just outside the cutoff (Figure S4(a) and (b)), and may provide a useful restraint.

FMN riboswitch (RFN element): RF00050 (112nt, 4const)

RF00050 is a complicated collection of short secondary structure segments but is essentially also a long bent-over hairpin with a T-shaped embellishment around which the rest of the molecule wraps and to which it is tethered by three separated groups of non-local restraints. (Figure S3(c) and (d)). The strongest of these is well placed to restrain the more distal stemloop to the T-shape and indirectly back to the trunk.

Lysine riboswitch: RF00168, (174nt, 4const)

Topological complexity is increased with RF00168 although it can still be viewed as a basic Y-shape but with a pair of stemloops added like a T-shape at the top of the trunk. As with some of the simpler molecules encountered above, predicted contacts cross-link the branches of the ”Y” but the ”T” is unrestrained. The three predicted non-local contacts correctly identify the pseudo-knot seen in the known structure but these effectively only constitute a single non-local tether. (Figure S4(c) and (b)).

Cobalamin riboswitch: RF00174 (329nt, 4const)

The complex structure of RF00174 contains several segments of secondary structure, most of which are well predicted by Gremlin (but not so well by RNAfold), with the exception of the stemloop at the $5'$ end — for the simple reason that all the sequences in the alignment are truncated by 22 bases. There are two regions of non-local contacts, one being a single base and the other a run of three bases. There is no restraint on the long extended hairpin or between two loops that make a form a base paired run involving the C/G rich sequence 75–80/194–200. The latter range falls in the truncated terminal region and would otherwise have provided a powerful restraint on the fold (if predicted). Lacking this important restraint, the results for RF00174 were not evaluated below.

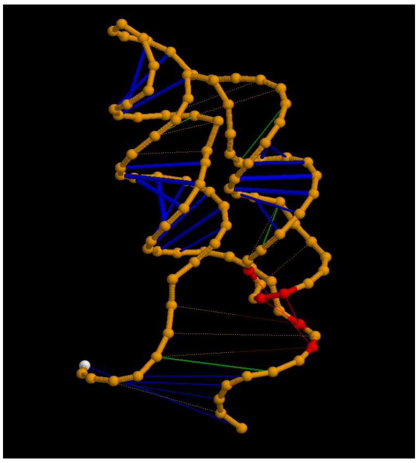

(a) RF01831 backbone

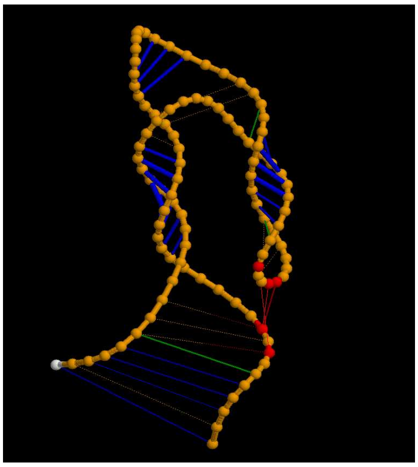

(b) RF01831 smoothed

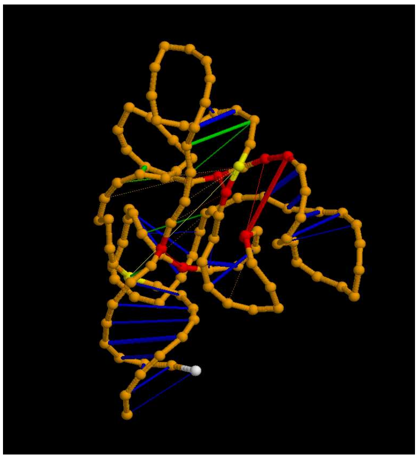

(c) RF00050 backbone

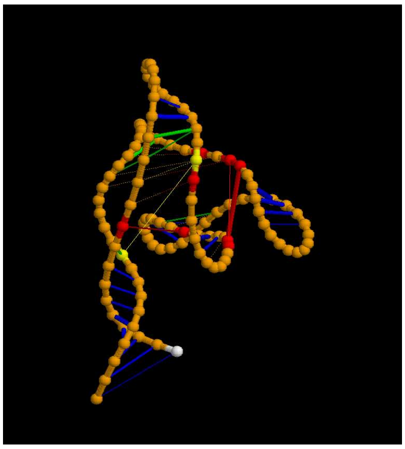

(d) RF00050 smoothed

Figure S3: **RF01831 (THF riboswitch) and RF00050 (FMN riboswitch)** phosphate backbones are shown in panels (*a*) and (*c*) with smoothed backbones in panels (*b*) and (*d*), respectively. Restraints are coloured as in Figure 2.

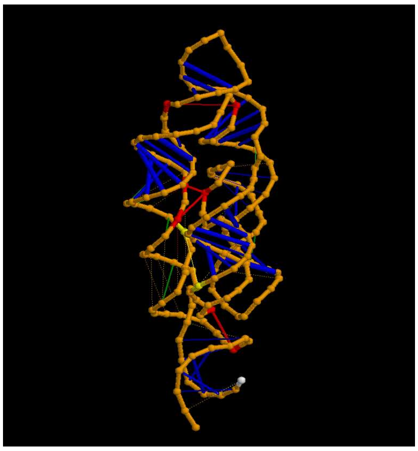

(a) RF00380 backbone

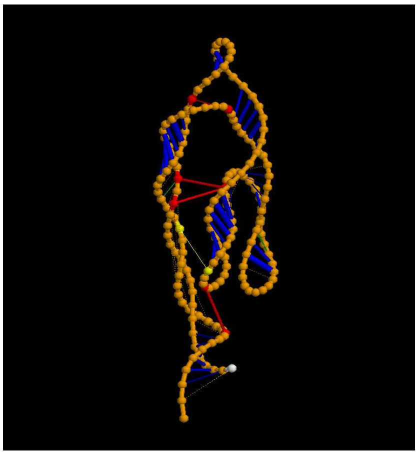

(b) RF00380 smoothed

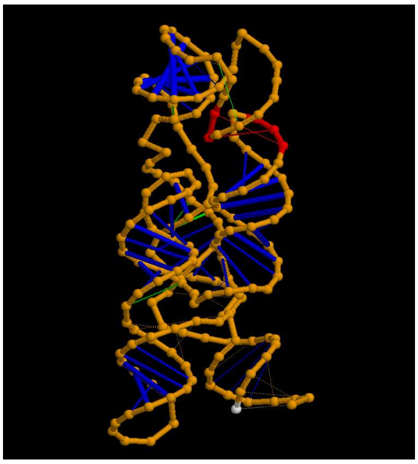

(c) RF00168 backbone

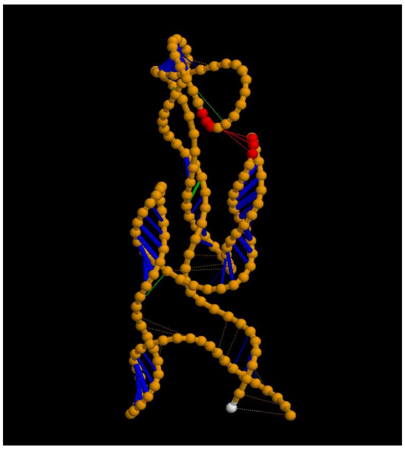

(d) RF00168 smoothed

Figure S4: **RF00380 (ykoK leader) and RF00168 (Lysine riboswitch)** phosphate backbones are shown in panels (*a*) and (*c*) with smoothed backbones in panels (*b*) and (*d*), respectively. Restraints are coloured as in Figure 2.

SAM riboswitch (S box leader): RF00162 (94nt, 6const)

This smaller structure can be viewed as a pair of secondary structures that have been mutually inserted into each other, as in a Holliday junction. (The cross-over point lies in the centre of Figure S5(a) and (b)). The end of one stemloop has four non-local restraints correctly predicted as forming a pseudoknot while two additional contacts cross-link the $5'$ end of the pseudoknot back towards the termini.

glmS glucosamine-6-P activated ribozyme: RF00234 (141nt, 6const)

Similarly, RF00234 also contains a correctly predicted pseudoknot and a few additional isolated cross-links elsewhere. This is the only molecule considered so far in which the terminal segments are not base paired, however, the predicted non-local contacts serve to tether the termini to the core.

Group I catalytic intron: RF00028 (246nt, 6const)

The larger RF00028 can be viewed as three domains of secondary structures in a ”W” like arrangement and although the termini are not base paired, they fall in the same domain. There are a number of highly ranked non-local predicted restraints but the majority of these either fall within a domain or at the base of the ”W” and so provide little restraint on the loops that lie away from the base. (Figure S6(a) and (b)).

Bacterial RNase P class A: RF00010 (347nt, 5const)

At almost 350 bases, RF00010 has a complicated structure but can be viewed as two domains with an additional isolated long stemloop (top right in Figure S6(c) and (d)). In proportion to its size, RF00010 has relatively few non-local predicted restraints and these fall into only two groups. One of these is a pseudoknot that tethers the end of the isolated stemloop and the other forms a cross-link at the

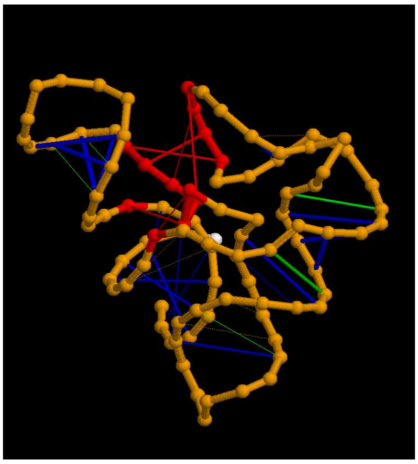

(a) RF00162 backbone

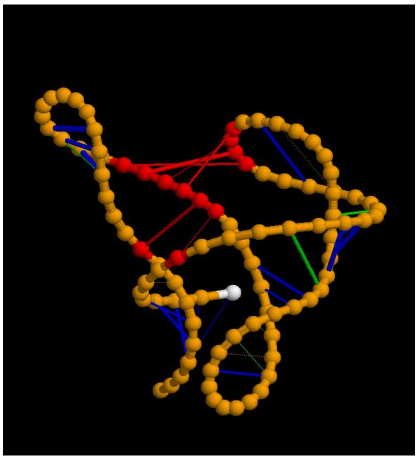

(b) RF00162 smoothed

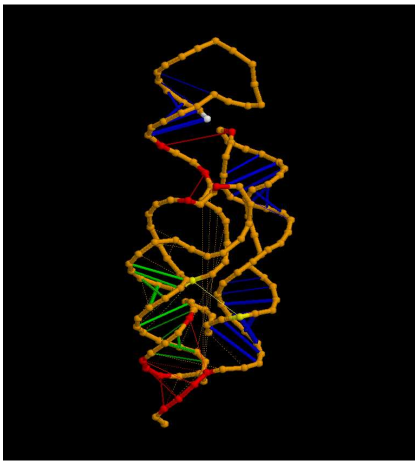

(c) RF00234 backbone

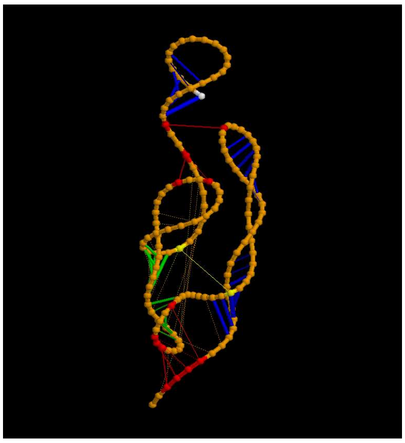

(d) RF00234 smoothed

Figure S5: **RF00162 (SAM riboswitch) 'and RF00234 (glmS glucosamine-6-phosphate activated ribozyme)** phosphate backbones are shown in panels (*a*) and (*c*) with smoothed backbones in panels (*b*) and (*d*), respectively. Restraints are coloured as in Figure 2.

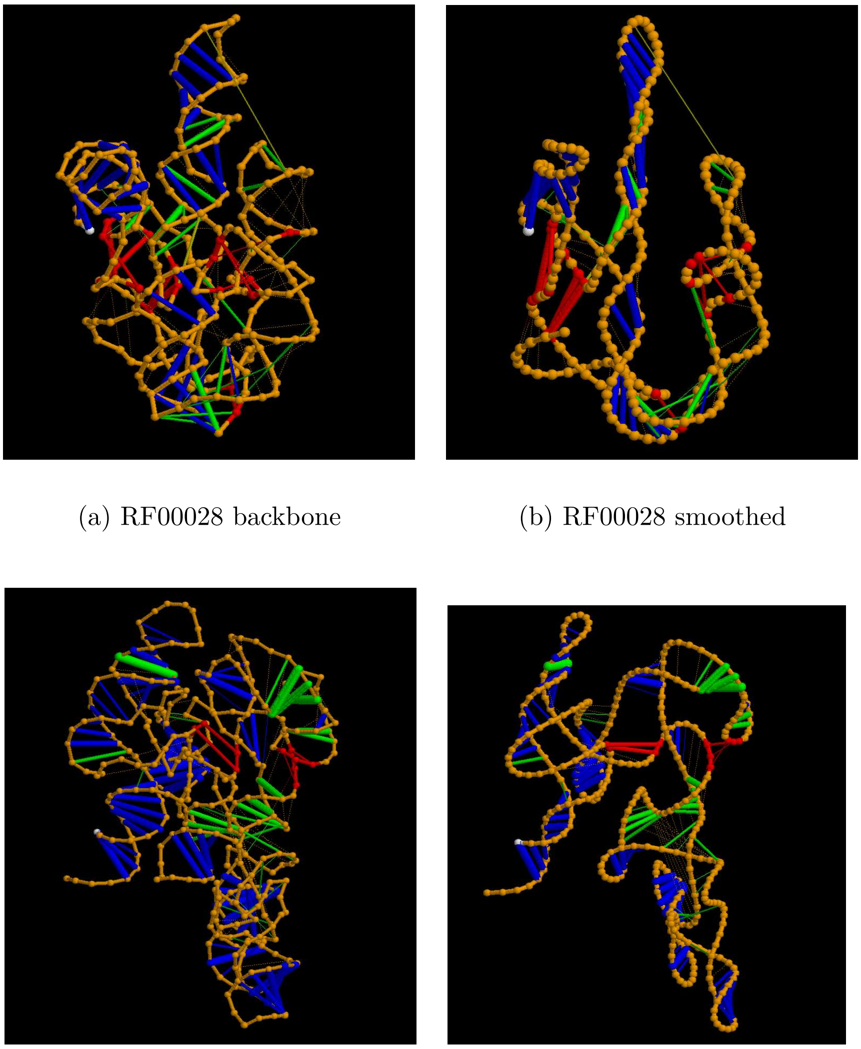

(a) RF00028 backbone (b) RF00028 smoothed

(c) RF00010 backbone (d) RF00010 smoothed

Figure S6: **RF00028 (Group I catalytic intron) and RF00010 (Bacterial RNase P)** phosphate backbones are shown in panels (*a*) and (*c*) with smoothed backbones in panels (*b*) and (*d*), respectively. Restraints are coloured as in Figure 2.

base of the same stemloop. There are no predicted cross-links between the two major domains.

Testing the perturbation level

A Single restraint

For each molecule, a simulation was run ten times with `SimRNA` and `SimGen` applying increasing levels of perturbation in separate runs over five levels. (See Methods section for details). With no applied perturbation in `SimGen`, the structures slightly drift away from the native structure due to errors in applied restraints and the ”correction” of non-ideal steric clashes, bond and angle geometry in the native molecule (which apply also when the observed base-pairing is used). After 1000 cycles, these effects lead to a deviation of around 5Å from the native structure. For `SimRNA`, the starting run-length was 1000 cycles, also giving a slight deviation from the native structure on the first test.

Plotting the degree of deviation with increasing levels of perturbation (Figure SS8), it can be seen that for three of the molecules, RF00167, RF00059 and RF00017, this follows an almost linear increase in mean RMSD from 5 to 15Å (for the standard RMSD measure, with the distance-based RMSD measures running at a slightly lower level). Such behaviour was expected for RF00059 and RF00017 where the restraint was poorly placed to restrain the overall fold and the curves for each set of restraints: observed base pairs (green), predicted base pairs (red) and predicted contacts (blue) follow much the same curve. The late separation seen for RF00059 using the dRMSDmax measure may result from a fortuitous prediction error that provides some restraint between the two stemloops. (Yellow in Figure S2(a)), which may also account for wider separation seen in the `SimRNA` curves (dashed lines in Figure S8(b)).

For RF01051, however, the curves are markedly different. The observed base pair restraints follow a typical denaturation curve and the predicted base pairs

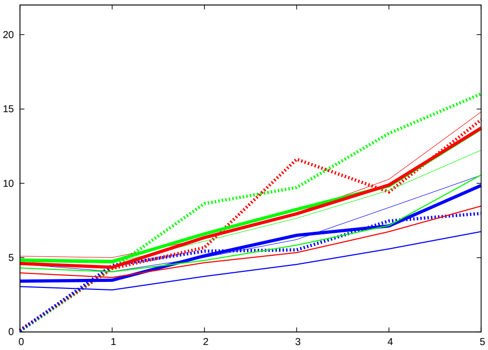

(a) RF00162

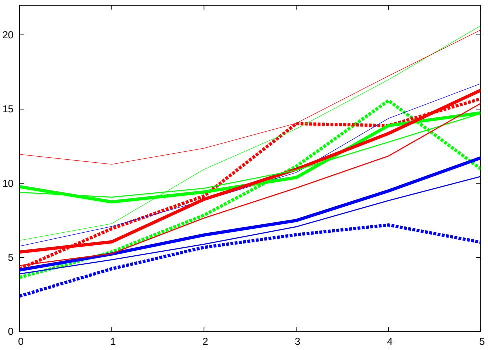

(b) RF00234

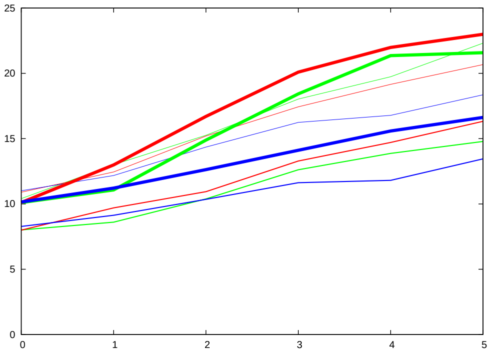

(c) RF00028

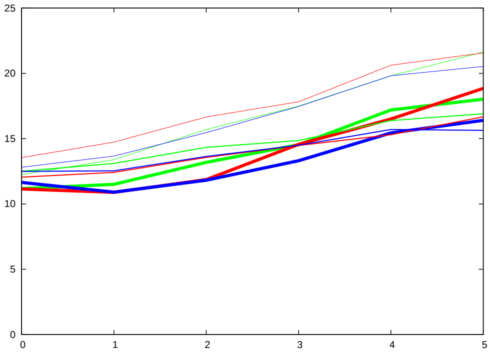

(d) RF00010

Figure S7: **Simulated denaturation curves with several restraints**. The mean RMSD value (Y-axis, Å) over ten models is plotted against the degree of random perturbation applied during the simulation (X-axis, arbitrary units). (See legend to Figure SS8 for a full explanation).

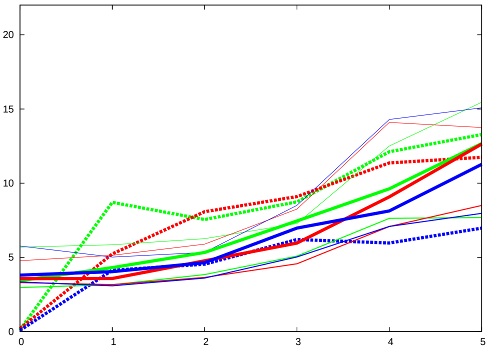

(a) RF00167

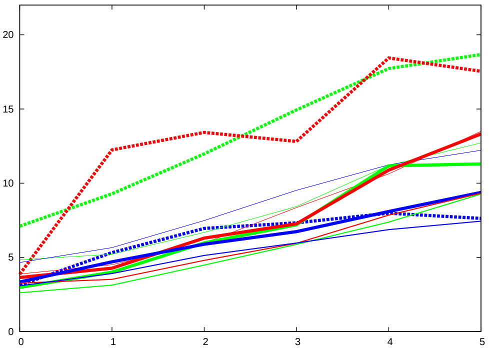

(b) RF00059

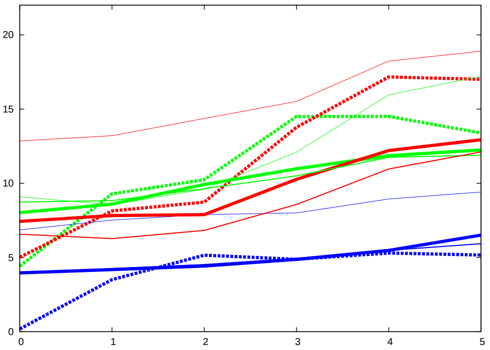

(c) RF01051

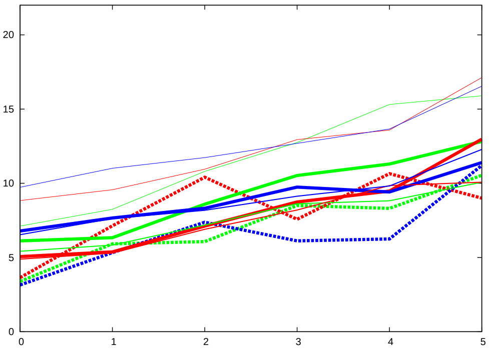

(d) RF00017

Figure S8: **Simulated denaturation curves with one restraint**. The mean RMSD value (Y-axis, Å) over ten models is plotted against the degree of random perturbation applied during the simulation (X-axis, arbitrary units). Three variants of the RMSD measure are plotted: thin lines = the standard (superposition) RMSD to the native structure, averaged over the models from ten simulations, medium lines = the distance-based dRMSD measure (also averaged over 10 models), thick lines = the maximum distance (dRMSDmax) seen over the 10 models. (See Methods section for details). The colours are: red = models made with the RNAfold predicted base pairs, green = models with the observed base pairs, blue = models with the predicted contacts from a correlated mutation analysis. Full lines are data from `SimGen` models and dashed lines are from `SimRNA` models.

start at a high RMSD level (due to prediction errors) and get worse. By contrast, the single non-local predicted contact held the structure below 10Å RMSD and around 5Å dRMSDmax even under the strongest disruption applied with both simulation methods (Figure S8(c)).

A similar behaviour had been expected for RF00167 and for the `SimRNA` results the restraint held the models 5Å closer to the native conformation than their unrestrained counterparts. However, with `SimGen` little difference was observed between the restraint sets. To check if this was due to a failure to maintain the restraint, the mean separation of the restrained pair of phosphates was compared across the three sets. The predicted contact was maintained at 17Å (a typical base pairing distance) whereas in the two sets where the pair is not restrained, the mean separation (over the ten models) was over 30Å in both. Visual examination of the models suggested that there was sufficient flexibility between the short double-strand sections to allow the two distal stemloops (Y-shape branches) to twist around each other while still maintaining the contact distance.

A few restraints

The single pseudo-knot restraint in RF01831 leads to a dramatic reduction in the RMSD of the models using predicted contacts with both simulation methods. This is complicated, however, by the flexibility of the single stranded termini that give rise to high RMSD values. The effect is reduced in the plots based on the dRMSD measures but these still show a good separation between the predicted contacts and the other two restraint sets. (Figure S9(a)). A similar but smaller effect is seen with RF00050 with respect to the observed base pair set. (Green and blue bold traces in Figure S9(b)).

By contrast, RF00380 and RF00168 show only a small drop with the application of the predicted contacts but with an increased effect seen in the latter molecule with the dRMSDmax measure using the `SimGen` method (Figure S9(c)

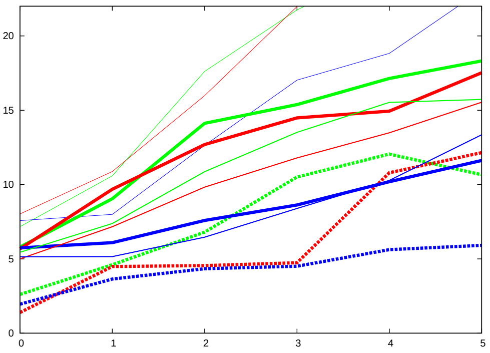

(a) RF01831

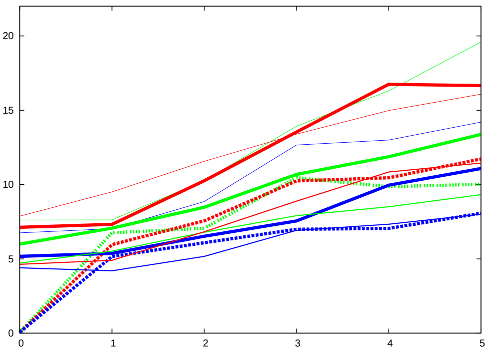

(b) RF00050

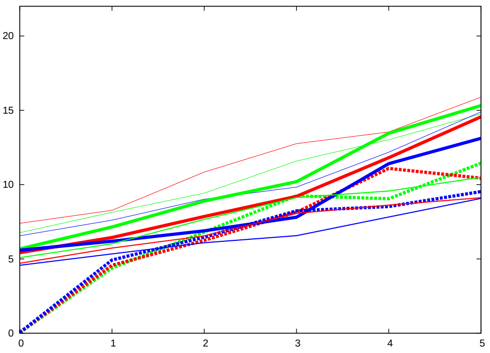

(c) RF00380

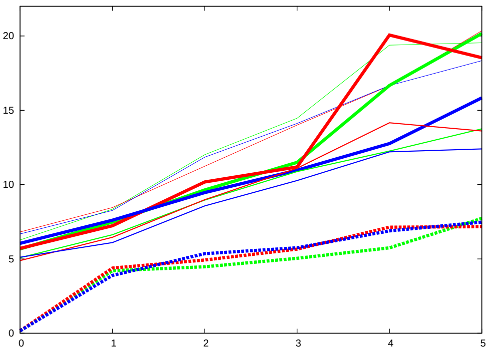

(d) RF00168

Figure S9: **Simulated denaturation curves with a few restraints**. The mean RMSD value (Y-axis, Å) over ten models is plotted against the degree of random perturbation applied during the simulation (X-axis, arbitrary units). Full lines are data from `SimGen` models and dashed lines are from `SimRNA` models. (See legend to Figure SS8 for a full explanation).

and Figure S9(d)). Both these molecules, however, have unrestrained stemloops and to see if these were masking the contribution of the restraints in part of the molecule, the mean RMSD (over the 10 models) for each position was plotted (Figure SS10). In the trace for RF00380, it can be seen that the unrestrained loop (positions 50–60) remains as high as with the local base pair sets of restraints, whereas in the restrained loop (80–90) the deviation is much lower (Figure S10(a)). Similarly with RF00168, the pair of unrestrained stemloops (in the T-shaped embellishment) at positions 120–130 and 145–155 are unaffected whereas the branches of the main Y-shape are well restrained for the shorter stemloop (90–100) and to a lesser degree for the longer branch (40–50). (Figure S10(b)).

The tighter restraint of the RF00168 molecule by the `SimRNA` method was the only situation where a distinct difference in behaviour was seen between the two simulation methods and to investigate this, the run-length of `SimRNA` was progressively increased. At 20,000 cycles, the first significant shift of a distal stemloop was seen giving an RMSD of 14Å. This uncharacteristic stability may simply be a consequence of size as this was the largest molecule run with `SimRNA`. With the predicted contacts active, the RMSD was half this value, equivalent to the `SimGen` result.

Several restraints

RF00162 shows a slightly greater retention of structure under the predicted restraints compared with the base pair sets and approaches a 5Å difference by the conventional (Euclidean) RMSD measure and only slightly less than this by the distance-based RMSD measures (Figure S7(a)). Plotting the degree of change with sequence, the largest effects with `SimRNA` were associated with loop that forms the pseudo-knot. With `SimGen`, however, the motion of the termini and the loop at position 50 were greater. (Figure S11(a)).

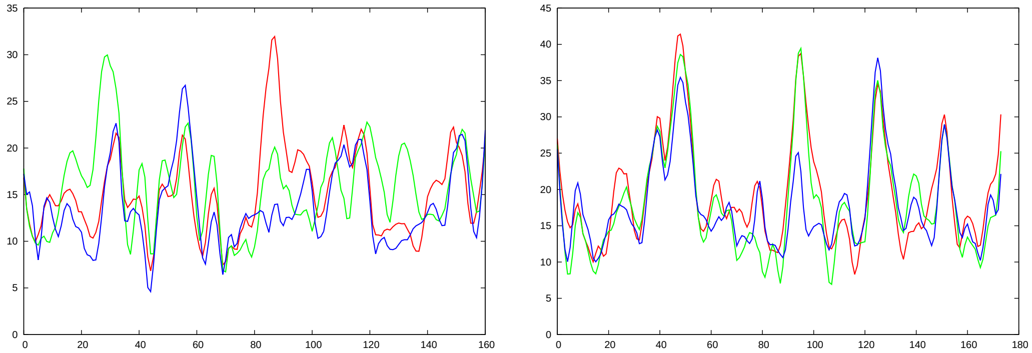

(a) RF00380 (b) RF00168

Figure S10: **Mean RMSD plotted along the chain**. The average RMSD from the native structure over ten `SimGen` models is plotted for each position along the chain for: (*a*) RF00380 and (*b*) RF00168. Different colours represent the three restraint sets as: red = predicted base pairs, green = observed base pairs and blue = predicted contacts.

Despite having a similar disposition of restraints, RF00234 shows a greater restriction on its conformational freedom with the imposition of the predicted contacts. This is largely a result of the restraints applied to the termini, although additional restriction can be associated with the pair of restraints cross-linking the loop region around position 50. (Figure S7(b)).

For the largest pair of structures considered, RF00028 and RF00010, the disposition of non-local restraints was not favourable for restricting the overall fold. Despite this, RF00028 retained almost a 5Å lower deviation when the predicted contacts were applied. As can be seen from Figure S11(c), the largest restraint is experienced around 160 which is close to the ladder of 5 strong restraints. The other restrained region around position 75 is associated with the relatively local restraints.

A more global view of the changes can be seen by comparing the distance matrices for the models generated with predicted restraint sets to the distance matrix of the native structure (Figure SS12). While the local structure around the

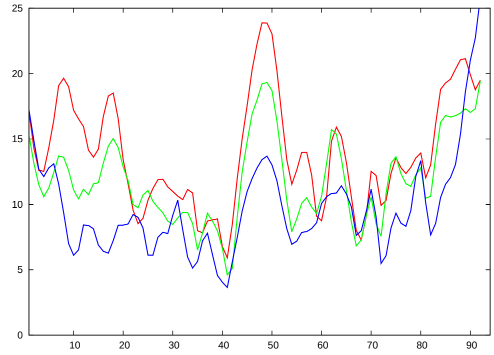

(a) RF00162

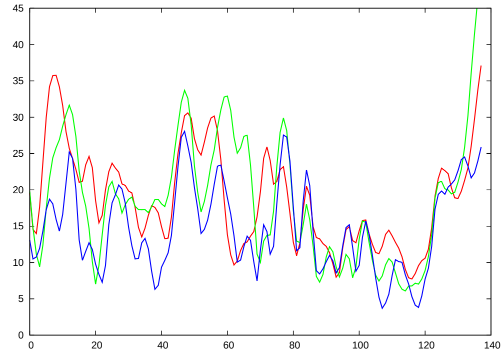

(b) RF00234

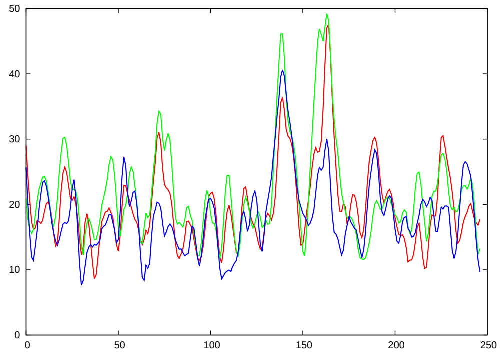

(c) RF00028

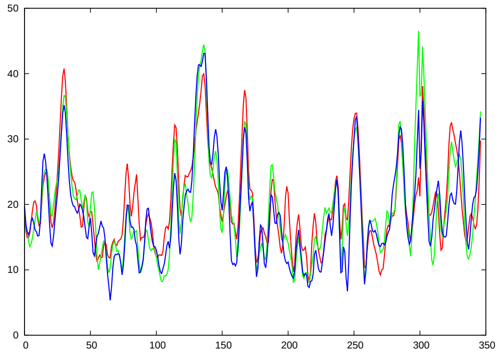

(d) RF00010

Figure S11: **Mean RMSD plotted along the chain**. The average RMSD from the native structure over ten `SimGen` models is plotted for each position along the chain for: (*a*) RF00162, (*b*) RF00234, (*c*) RF00028 and (*d*) RF00010. Different colours represent the three restraint sets as: red = predicted base pairs, green = observed base pairs and blue = predicted contacts.

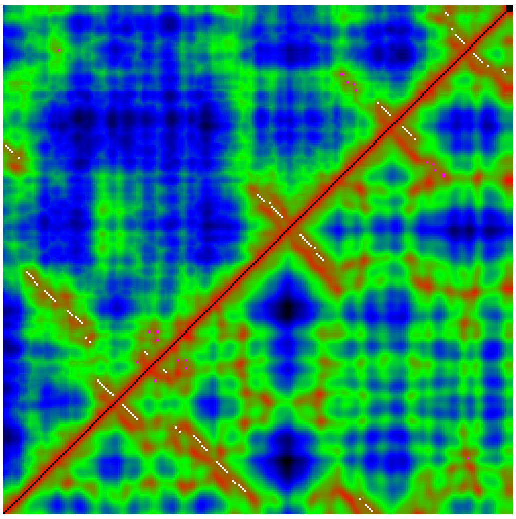

(a) contacts/native

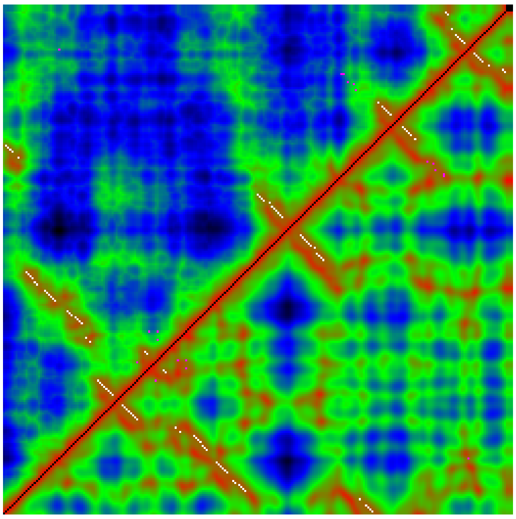

(b) basepair/native

Figure S12: **Distance matrices for RF00028**. The pairwise distances between phosphates in RF00028 is plotted as a distance matrix coloured red for the closest pairs through green and blue to black for the most widely separated pairs. The white dots mark local base paired positions and a few magenta dots mark non-local pairs. The lower-right half of the matrix shows the native distances and the top-left show distances from (*a*) the maximum distance seen in the ten models generated using the Gremlin predicted contact restraint set and (*b*), the RNAfold predicted base pairs.

base paired segments remain preserved in both predicted matrices (white dots), there is a considerable increase in the number of longer distances (blue/black).
